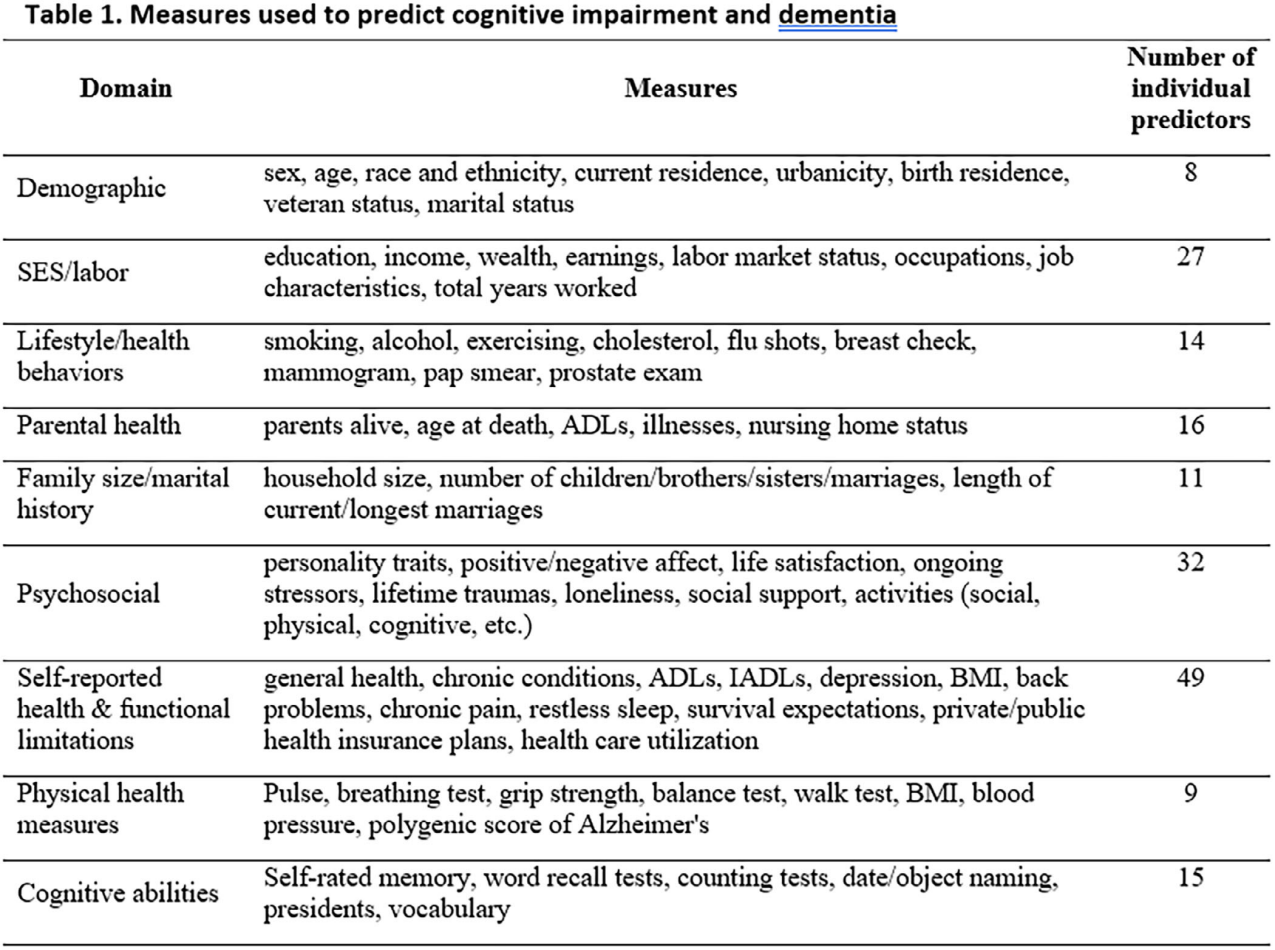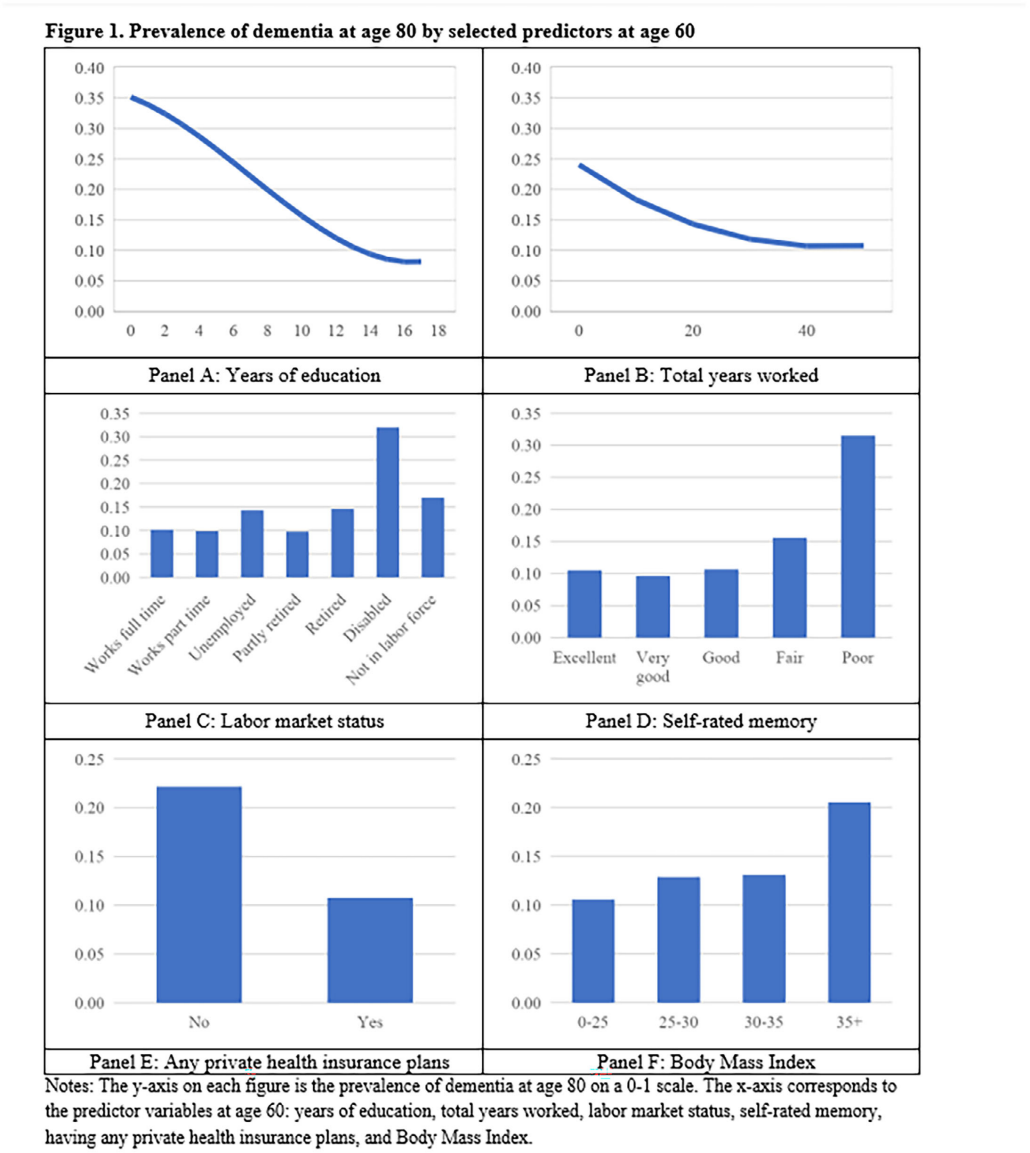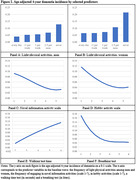# Identifying early predictors of cognitive impairment and dementia in a large nationally‐representative US sample

**DOI:** 10.1002/alz.089619

**Published:** 2025-01-09

**Authors:** Peter Hudomiet, Michael D Hurd, Susann Rohwedder

**Affiliations:** ^1^ RAND, Santa Monica, CA USA; ^2^ Netspar, Tilburg Netherlands; ^3^ National Bureau of Economic Research, Cambridge, MA USA

## Abstract

**Background:**

Dementia impacts a large and growing number of older adults in the US, and the total impact of disease is costly to individuals and society. Though many risk factors have been identified, accurately predicting future dementia remains difficult. This study aims to identify early predictors of cognitive impairment and dementia using a large US sample.

**Method:**

We evaluated the predictive power of 181 dementia risk factors using a recently developed and validated probabilistic measure of dementia and cognitive impairment, available on 97,629 person‐year observations in the nationally representative longitudinal Health and Retirement Study in the US (Table 1). The project predicted two types of outcomes: the two‐year or four‐year incidence of dementia or cognitive impairment; and the prevalence of dementia or cognitive impairment at age 80 based on the characteristics of individuals at age 60.

**Result:**

In line with prior literature, physical health, a prior stroke, cognitive abilities, ADL and IADL functional limitations, and genes strongly predicted future incidence and prevalence of cognitive impairment and dementia. Our statistical models identified additional predictors that have received less attention. For example, dementia prevalence at age 80 was 9.9 percentage points higher (20.5% vs. 10.6%) among those with a BMI above 35 (vs. BMI 0‐25) at age 60, and this differential reduced to 4.2 ppts but remained statistically significant when all other predictors were controlled. Other factors associated with a higher chance of having cognitive impairment or dementia in the future were: being born in the South, not having a private health insurance plan at age 60, working fewer years, diabetes at age 60, never exercising, scoring lower on various physical performance measures (e.g. pulmonary function, grip strength, walking speed, balance tests), being less conscientious, and lower engagement in hobbies and novel information activities (Figures 1 and 2).

**Conclusion:**

The sharpened list of dementia risk factors can be used to raise awareness and target resources for prevention and early detection in the presence of health system capacity constraints. These results are suggestive that some behavioral changes and interventions might slow cognitive decline.